# Utilization of red and yellow *Coffea arabica* var. Caturra pulp: macronutrient analysis, carotenoid extraction, and encapsulation for dairy product enrichment

**DOI:** 10.3389/fnut.2023.1231049

**Published:** 2023-08-31

**Authors:** Elkin Rojas-Orduña, María Hernández-Carrión, Juan David Gómez-Franco, Carlos-Eduardo Narváez-Cuenca, Andrea del Pilar Sánchez-Camargo

**Affiliations:** ^1^Group of Product and Process Design, Department of Chemical and Food Engineering, Universidad de los Andes, Bogotá, Colombia; ^2^Food Chemistry Research Group, Departamento de Química, Facultad de Ciencias, Universidad Nacional de Colombia, Bogotá, Colombia

**Keywords:** β-carotene, encapsulation, ultrasound-assisted extraction, solid–liquid extraction, coffee pulp, dairy product

## Abstract

This study aimed to investigate the macronutrient and carotenoid content of red and yellow *Coffea arabica* var. Caturra pulp, a by-product of coffee processing in Colombia. The study employed ultra-sound-assisted extraction (UAE) to extract carotenoids, and a 2^3^ factorial design was used to evaluate the effects of pulp color, biomass-solvent ratio, and solvent mixture composition on carotenoid content and extraction yield. The condition that provided the highest carotenoid extraction was further encapsulated by spray drying and added to a dairy product. The results showed that coffee pulp has significant dietary fiber content and high levels of carotenoids, with yellow pulp having a higher content than red pulp. Lutein isomers and lutein esters were the most abundant carotenoids found in both red and yellow coffee pulp. The highest carotenoid extraction was achieved using a 1:40 (g/mL) biomass:solvent ratio and a 20:80% v/v Ethanol:Ethyl Acetate solvent mixture for the yellow pulp. The carotenoid extract also demonstrated high encapsulation efficiency (46.57 ± 4.03%) and was found to be stable when added to a fermented milk product. This study presents an alternative solution for utilizing coffee by-products in Colombia, which could positively impact the families of over half a million Colombian coffee producers.

## 1. Introduction

Coffee is a widely consumed beverage globally, produced from two species, *Coffea canephora* (Robusta) and *Coffea arabica* (Arabica). Arabica species is considered to have superior sensory qualities and commands a higher market price as compared to Robusta (1). Indeed, the Arabica coffee production for 2021/22 was higher (87.4 million 60-kilogram bags) than the Robusta coffee production (79 million 60-kilogram bags) (2). The main appeal of coffee lies in its stimulating effect; however, it has also been linked to various health benefits including reducing the risk of diabetes (3), hypertension (4), stroke (5), and coronary heart disease (6).

Currently, Colombia is the third largest producer of coffee with nearly half a million coffee-producing families, in an area of approximately one million hectares planted. For 2021, coffee production in Colombia was 11.1 million 60-kilogram bags (7). The varieties that are mainly cultivated in Colombia are the Típica and Bourbon varieties, although the Castillo and Caturra varieties are prominent as well. The Caturra variety is distinguished by its ability to yield a high volume of coffee production, generate medium size beans, and produce a superior cup in tasting evaluation (8). It has the disadvantage, however, of being susceptible to infection by the rust fungus (*Hemileia vastatrix*), which mainly affects its leaves and hinders normal plant metabolism (9).

**Figure 1:**
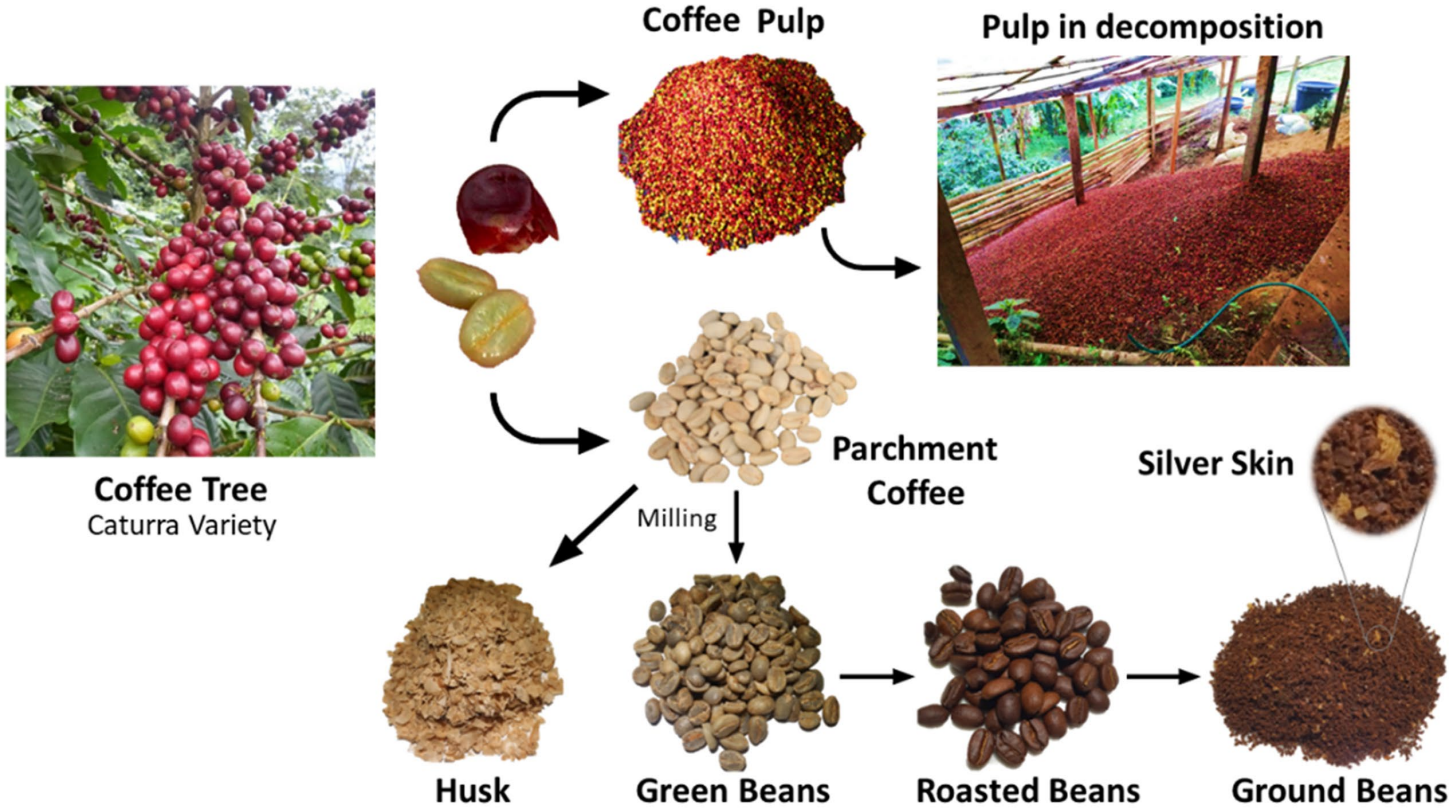
Plant, cherry, and coffee processing residues in Comercializadora Tint Café S.A.S., Colombia.

The coffee cherry is comprised of six main parts: the skin, pulp, mucilage, parchment, silverskin, and seed. Figure 1 displays photographs of the fractions obtained in the coffee processing by a local producer. During coffee processing, around 50% of the cherry’s total weight is discarded as waste (10). Thus, approximately 1.89 million tons of coffee pulp are produced annually in Colombia. This by-product is poorly disposed and often become a significant source of water and soil pollution in coffee-growing regions (11). In the last decade, the use of coffee processing by-products has been studied for their potential role in the energy sector, including generation of gas and biofuels, in the agro-industry as animal feed, and particularly in the food industry as a source of high-value added compounds (11, 12). Despite the long-standing interest in extracting valuable compounds from coffee waste (11–13), large-scale extraction has not yet been economically viable due to the low concentration of these compounds in the raw material (13).

The presence of compounds of interest, such as caffeine, in coffee pulp represents a potential source of raw material for extraction, particularly due to the recent surge in demand for caffeine in the production of energy drinks, cosmetics, and pharmaceuticals (14). There is also a growing interest in removing caffeine and certain phenolic compounds from coffee pulp to mitigate the adverse effects they may have on animals when utilized in animal feed production (15). Carotenoids are another group of molecules of interest that also can be extracted from coffee by-products, attracting the attention of researchers (13, 16–21). Carotenoids are naturally occurring organic pigments found in plants, algae, fungi, and bacteria (22). Their antioxidant properties and provitamin A nature make them valuable for use in the food and cosmetic industries (22). The carotenoids profile of coffee pulp depends on the variety and color of the coffee. In the case of the red and yellow pulps of the Caturra variety, they mainly contain lycopene, β-carotene, γ-carotene, zeaxanthin, and lutein (16).

The prospect of extracting carotenoids from coffee industry by-products becomes further attractive as it can be accomplished by using non-conventional methods. These alternative methods offer advantages over conventional methods in terms of reduced environmental impact (23, 24). Ultrasound-assisted extraction (UAE) is a widely studied non-conventional extraction method that employs acoustic cavitation to break down the cell wall of the biomass and enhance the interaction between the solvent and the targeted compounds. This technology is frequently used for the extraction of phenolic compounds and antioxidants from plant biomasses (25–27).

The limited incorporation of carotenoids into food is due to their chemical instability caused by factors such as temperature, light, and oxygen, as well as the non-polar and unsaturated structure of their molecules (28). Microencapsulation can mitigate these effects by encasing the carotenoids within an edible, solid polymeric matrix through freeze-drying or spray-drying processes (29, 30). Microencapsulation enhances the shelf life of the carotenoids and enables their addition to food for health benefits through ingestion (31). For instance, Rocha et al. (32) carried out an encapsulation of lycopene using modified starch as wall material and found that the encapsulation effectively provides greater stability to the molecules as compared to those that did not receive such treatment. For their part, Šeregelj et al. (33) developed an encapsulated of sunflower oil-diluted carotenoid extract obtained from red pepper waste using whey protein as wall material. This encapsulated extract was added to a dairy product, and the stability of those compounds and the rheological changes of the food were studied. Other successful cases of carotenoid encapsulation application in food products are included in a review by Roll-Zimmer et al. (34).

In this sense, the dairy industry, known for its easy accessibility to consumers and high nutritional value of its products (35), holds significant potential for the development of functional foods, and as an example, yogurt being the standout fermented dairy food product due to its benefits for the intestinal microbiota and overall nutrient content (36). With the growing consumption of fermented dairy products worldwide, there is a significant opportunity to create high-value functional foods (37).

With this context in mind, the objective of this study was to evaluate the composition of macronutrients, carotenoid content, and compound profile present in red and yellow coffee pulp from *Coffea Arabica* var. Caturra. Additionally, the aim was to obtain enriched carotenoids by assessing the factors that affect ultrasound-assisted extraction, encapsulating the optimal extract through spray drying, and incorporating it into a dairy product. The resulting dairy product was characterized in terms of color, stability, and rheological properties. To the best of our knowledge, this study represents the first successful application of carotenoids derived from coffee industry by-products in a dairy product.

## 2. Materials and methods

### 2.1. Sample and reagents

In this study, Comercializadora Tint Café S.A.S, Villeta, Colombia, supplied coffee pulp samples of both colors (red and yellow). β-Carotene of 97.0% (*w/w*) purity was used from Sigma-Aldrich (product number 7235-40-7). The chemicals 2,2-diphenyl-1-picrylhydrazyl (DPPH), 2,2′-Azinobis (3-ethylbenzothiazoline-6-sulphonic acid) diammonium salt (ABTS), and anhydrous caffeine (C_8_H_10_N_4_O_2_), were also used from Sigma-Aldrich. Absolute ethanol (C_2_H_5_OH, 99.8% *v/v*), ethyl acetate (C_4_H_8_O_2_, 99.5% *v/v*), acetone (C_3_H_6_O, 99.5% *v/v*), hydrochloric acid (HCl, 37.0% *v/v*), and sodium hydroxide (NaOH, 98.0% *w/w*) were obtained from Panreac (Gmbh). For the determination of total dietary fiber (TDF), insoluble dietary fiber (IDF), and soluble dietary fiber (SDF) heat-stable α-amylase, protease, and amyloglycosidase (Sigma® Life Science) were utilized for enzymatic digestion. Anhydrous disodium hydrogen phosphate (Na_2_HPO_4_, 99.0% *w/w*) and anhydrous sodium dihydrogen phosphate (NaH_2_PO_4_, 99.0% *w/w*) from Panreac were used for preparing the phosphate buffer in TDF, IDF, and SDF quantification. For the extract dilution a Don Olio (Colombia) sunflower oil was used. As wall materials, arabic gum from CIACOMEQ S.A.S. and maltodextrin from Cimpa S.A.S. were used.

### 2.2. Sample preparation

The coffee pulp (red and yellow) was washed, disinfected (100 mg/L sodium hypochlorite), and stored at −18°C. The pulp was divided into two parts, one was dried by forced convection and the other one was lyophilized. The forced convection dehydration process took place in a tray dryer (UOP8-MKIII Armfield, UK) at 40°C for 24 h under atmospheric pressure. For freeze-drying, the samples were frozen at −80°C in an ultra-freezer (Haier Biomedical, UK) prior to the start of the lyophilization process at −40°C for 72 h and 0.086 bar in a freeze-dryer (FreeZone 6 Liter Benchtop, Labconco Corp., KS). Both the lyophilized and forced convection dehydrated samples were ground in a rotary blade mill (Pulverisette 19, Fritsch Comp, Germany). The ground samples were sieved using Tyler series flatbed sieves (Tamiz Standard, Colombia). The particle size fraction for extraction experimental design was between 250 and 425 μm.

### 2.3. Chemical characterization of coffee pulp

The proximate composition analyses were conducted using the techniques described in the AOAC (38) on pulp that was dehydrated by forced convection. Moisture content was determined by oven-drying at 105°C (AOAC 930.15); total fat was determined using the Soxhlet method with hexane (AOAC 991.36); crude protein was quantified using the microKjeldahl procedure (AOAC 960.52); ash content was determined by incineration in a muffle furnace at 550°C (AOAC 942.05), and total dietary fiber (TDF), insoluble dietary fiber (IDF), and soluble dietary fiber (SDF) were quantified using the enzymatic-gravimetric method (AOAC 985.29). The caffeine content was determined by high performance liquid chromatography (HPLC), following the method described by the International Standard ISO 20481:2008 (International Organization for Standardization ISO, 2008). For the calibration curve, anhydrous caffeine solutions with concentrations between 1 and 100 mg/mL (five data points, *R*^2^ = 0.9999) were prepared. All mentioned determinations were performed for each pulp color in triplicate.

### 2.4. Total carotenoids content by UV–vis spectroscopy

The carotenoid compounds present in the pulp coffee samples were extracted by conventional extraction (CE) following the methodology described by Biswas et al. (39), with some modifications. One gram of each pulp color and each of the two drying methods was weighed and placed in a 50 mL-Falcon tube. Successive extractions (until the extract was colorless) were conducted using 10 mL of cold acetone and a Dispermat^®^ homogenizer (DISPERMAT^®^ LC Dissolver, Vma-Getzmann Gmbh, Germany) with a 20 mm light disc set at 10,000 rpm for 5 min. Then, the extracted material was centrifuged (CL30R, Thermo Fisher Scientific, MA, USA) at 4,300 rpm for 8 min at 0°C, filtered with qualitative filter paper (65 g/m^2^), and collected in another Falcon tube. The extract was then adjusted to a 50 mL with cold acetone and the absorbance was measured at 454 nm using an UV–vis spectrophotometer (Thermo Scientific Genesys 20^®^, USA). Total carotenoids content (TCC) was calculated using a calibration curve of *all-trans*-β-carotene (10–80 μg/mL, eight data points, R^2^ = 0.9975). Acetone was used as a blank. Results were expressed as mg *all-trans*-β-carotene equivalents/g sample DW. The procedures were performed in triplicate.

### 2.5. Carotenoids extraction and profiling by RP-UHPLC-DAD

Extraction was performed using a mixture of solvents containing butylated hydroxyanisol (BHA). For this, 50 mg of each sample (freeze-dried red or yellow pulp) was extracted with 1 mL acetone:methanol (2:1, *v/v*) containing 0.5% (*w/v*) BHA and 0.5 mL hexane during 20 min at 0°C in an ultrasonic bath (Branson 1510, Danbury, CT, USA). The mixture was centrifuged (14,000 rpm; 10 min; 4°C), the supernatant was collected and stored at 4°C. The pellet was re-extracted two times. The three extractions yielded more than 95% carotenoids extraction as evaluated at 450 nm. The three extracts were pulled together, and a liquid–liquid partitioning was done with 4.5 mL aqueous 1 M sodium chloride. The organic layer was dried under nitrogen gas. The dried extract was re-disolved in 1 mL acetone:methanol (2:1, *v/v*) containing 0.5% (*w/v*) BHA and analyzed by UHPLC. Chromatographic analyses were performed on a UHPLC 3000 DIONEX (Sunnyvale, CA, USA) equipped with a pump, auto-sampler, and a diode array detector (DAD). The extract (5 μL) was injected onto an Aquity UPLC BEH Shield RP18 column (150 mm × 2.1 mm, 1.7 μm, Waters, Milford, MA, USA). Chromatographic separation was carried out at 20°C. Elution was developed by a gradient of three solvent mixtures, solvent A: acetonitrile/triethylamine (100:0.25, *v/v*), solvent B: ethyl acetate/triethylamine (100:0.25, *v/v*), and solvent C: acetonitrile/water/triethylamine (50:50:0.25, *v/v/v*). The composition was modified during the chromatographic program by changing the proportions of solvents A and B, while solvent C was kept constant at 5%. The elution program was: 0–1 min (0–0% B), 1–13 min (0–30% B), 13–15.5 min (30–30% B), 15.5–17.5 min (30–0% B), 17.5–27 min (0–0% B). The flow rate was set at 0.250 mL/min. Detection was performed at 450, 470, 477, and 503 nm (40).

Identification was carried out by analyzing the order of elution, comparison with absorption spectra (λ_max_), and chromatographic characteristics of authentic and similar carotenoids, as well as reference parameters in the literature such as spectral fine structure (%III/II) and maximum *cis* intensity (II/B) (41). The % III/II ratio represents the correlation between the peak heights of the longest wavelength absorption band (III) and the middle absorption band (II). This calculation is performed by taking the minimum peak between the two bands as the baseline and multiplying it by 100. Additionally, the II/B ratio was determined by dividing the peak heights of the middle absorption band (II) by that of the *cis* peak, which appears in the UV region. Moreover, authentic standards of all-trans-lutein, all-trans-zeaxanthin, and all-trans-β-carotene were injected separately and co-injected with the extracts for comparative analysis. Each carotenoid was quantified using external analytical curves with six points (in duplicate) for *all*-*trans*-β-carotene (1–100 μg/mL, *R*^2^ = 0.9999). Only peaks with area higher than 2% of the total area as measured at 450 nm and those were resolution did not compromised tentative peak identification and quantification were analyzed. The limits of detection (LOD) and quantification (LOQ) were determined as three and ten times the standard deviation of the noise (blank), respectively. The LOD was 0.3 μg/mL and the LOQ was 1.0 μg/mL. Results were reported as the average of the four replicates performed (±standard deviation) in μg *all*-*trans*-β-carotene equivalents/g dry sample. No correction was made for differences in molar absorption coefficients or molecular weight.

### 2.6. Extraction of carotenoids using UAE

A factorial experimental design (2^3^) was proposed to optimize the UAE of carotenoids from coffee pulp. The effect of color pulp [red (−), yellow (+)], biomass:solvent ratio [1:20 (−), 1:40 (+) *w/v*], and the solvent composition, ethanol:ethyl acetate ratio [20:80 (−), 80:20 (+) %*v/v*] on (i) the content of carotenoids extracted from the biomass (CEB) [mg *all*-*trans*-β-carotene equivalents/g biomass dry weight], (ii) the extraction yield (EY) [%, g extract/100 g biomass dry weight], and (iii) the concentration of carotenoids in the extract (CCE) [mg *all-trans*-β-carotene equivalents/g extract dry weight], was investigated. The experimental design was developed in a setup with a tip sonicator (Sonics Materials VCX-750-220, Thermo Fisher Scientific, MA, USA), which included a 13 mm probe, operating at 35% amplitude and a pulse ratio of 20:40 s active-rest time for 30 min. Freeze-dried pulp coffee samples and solvent mixtures were placed in a light-protected Schott container and kept in an ice bath during the procedure. The extraction process was carried out in triplicate for each condition.

After extraction, the raw extract was centrifuged at 4,300 rpm for 8 min at 0°C. The resulting supernatant was filtered with 65 g/m^2^ qualitative filter paper and adjusted to 50 mL or 100 mL according to biomass:solvent ratio. The absorbance of each solution was measured at 454 nm and the CEB was calculated. For the carotenoids quantification, two calibration curves of *all-trans-*β-carotene were carried out: one for the mixture solvent ethanol:ethyl acetate 80:20% (*v/v*) (10–80 μg/mL, eight data points, *R*^2^ = 0.9992) and another one for ethanol:ethyl acetate ratio 20:80% (*v/v*) (10–80 μg/mL, eight data points, *R*^2^ = 0.9930). To determine the EY, the residue left after removing the solvent in a rotary evaporator (Yamato, RE801, USA) was weighed from 35 mL of extract. The CCE response variable was calculated as the ratio between the CEB and EY.

### 2.7. *In vitro* antioxidant capacity assays of carotenoid-enriched extract

The extract with the highest CCE value was evaluated on its *in vitro* antioxidant activity by the DPPH and the ABTS radical methods.

#### 2.7.1. Half maximal effective concentration (EC_50_) by DPPH radical

The protocol developed by Brand-Williams et al. (42), and detailed by Sánchez-Camargo et al. (43), was employed to determine the EC_50_ value. The concentration required for a 50% decrease in the initial activity of the DPPH radical was determined. A lower EC_50_ value is indicative of a higher antioxidant capacity. The experiments were done in triplicate.

#### 2.7.2. Trolox equivalent antioxidant capacity (TEAC)

The TEAC assay was performed following the ABTS radical method as described elsewhere and based on the procedure of Re et al. (44).

### 2.8. Encapsulation by spray drying of the optimal carotenoid-enriched extract

The concentrated extract with the highest CCE value was encapsulated following the methodology described by López-Bermudez et al. (45). For this purpose, the extract was re-diluted in a known volume (30 mL) of sunflower oil. The polymeric matrix was prepared using gum arabic and maltodextrin in water with a 1:3 (*w/w*) ratio wall materials:water. Both substances are classified in the Generally Recognized as Safe (GRAS) category by the U.S. Food and Drug Administration (FDA) (46, 47). The wall materials were hydrated separately before mixing using a mechanical stirrer (Hei-Torque, Heildolph, Germany) at 1,200 rpm for 5 min. The emulsion (O/W) was prepared by adding the enriched oil to the polymeric matrix in a ratio 2:1 (*w/w*) wall material:oil and continuing the stirring for 10 min more. Finally, homogenization was performed in a rotor-stator (SilentCrusher M, Heidolph, Germany) at 18,000 rpm for 5 min. The emulsion was fed to the spray dryer (B290, Buchi AG, Switzerland) with an inlet flow rate of 6.25 mL/min, using an inlet and outlet temperature of 140 and 110°C, respectively. A 0.7 mm diameter co-current spray nozzle with an air inlet of 473 L/h was used. Subsequently, the sample was stored in a desiccator at room temperature avoiding contact with light. The process was done in triplicate.

#### 2.8.1. Drying yield and encapsulation efficiency

On the one hand, the determination of the spray-drying process yield was accomplished by calculating the percentage of the mass of the powder obtained in relation to the mass of solids in the emulsion that was fed into the spray-dryer. This calculation was carried out using Equation 1, as described by Duran-Baron et al. (48). The procedure was carried out in triplicate.

(Eq. 1) $$DY\left(\%\right)=\frac{massoftotalpowderobtained\;\left(g\right)}{massofsolidsinfeedemulsion\;\left(g\right)}*100$$

On the other hand, the determination of encapsulation efficiency was conducted by calculating the ratio between the TCC of the powder (mg *all-trans*-β-carotene equivalents/g of powder obtained) and the TCC of the diluted extract (mg *all-trans*-β-carotene equivalents/ g of sunflower oil). To determine the TCC of the powder, the same method as described in Section 2.4 for UV–vis spectroscopy was utilized. The procedure was carried out in triplicate.

#### 2.8.2. Encapsulate characterization

The moisture content (MC) of the encapsulate was assessed using a moisture analyzer (Precisa, Series 330 XM, Switzerland) at 105°C until a constant weight was achieved. The results were reported as a percentage on a wet basis.

Water activity was measured using a water activity probe (HC2-AW, Rotronic AG, Switzerland). For that, 2 g of powder were used, and the result was reported in terms of water activity (a_w_).

The dissolution rate was determined by measuring the time taken to completely dissolve 2 g of the encapsulate in 50 mL of water, while being stirred at 900 rpm at room temperature. This method was previously described in the study conducted by López-Bermudez et al. (45).

The tapped density was determined according to the methodology described by Duran-Baron et al. (48), by compacting 5 g of encapsulate in a 25 mL graduated cylinder.

Provitamin A activity was calculated in terms of μg Retinol Activity Equivalents (RAE), in which a conversion factor of 1 μg RAE/6 μg β-Carotene equivalents was used (49). All measurements described under this sub-heading were performed in triplicate.

### 2.9. Formulation and development of the dairy product

To prepare the yogurt, 1 L of pasteurized whole milk from Cooperativa Colanta, Colombia was used and maintained at 42 ± 1°C throughout the fermentation process. The milk with a 250 mg capsule with 1 × 10^9^ CFU of starter culture of Bulgarian yogurt (Genesis Laboratories Ltd., Bulgaria) was allowed to reach a pH of 4.5 ± 0.1, and then it was cooled to 4°C. The encapsulated enriched-carotenoids extract was mixed with cooled yogurt in a proportion of 9.5% (*w/v*) to classify this dairy product as an “excellent source” of vitamin A according to Colombian regulation ‘Resolution 810 of 2021’ (50). This amount of encapsulated is the minimum necessary considering that the standard requires 15% of 800 μg RAE. The mixture was made in a Schoot flask with magnetic stirring for 20 min at 1,200 rpm. The mixing was done in triplicate for a fortified yogurt (FY), and a stirred control (SC) without encapsulated. Additionally, to consider the effect of the stirring on the rheological properties, a sample of the yogurt before agitation was taken as an unstirred control (UC).

### 2.10. Dairy product characterization

#### 2.10.1. Colorimetry index

The Colorimetry index (L*, a*, b*) of the FY and the UC was measured using a solid colorimeter (CR-20, Konica Minolta, Japan) with standard illuminant D65 and observer at 10° and the overall color difference against the control was calculated (Equation 2). The analyses were performed in triplicate.

(Eq. 2) $$\Delta E=\sqrt{{\left(L_{UC}^{*}-L_{FY}^{*}\right)}^{2}+{\left(a_{UC}^{*}-a_{FY}^{*}\right)}^{2}+{\left(b_{UC}^{*}-b_{FY}^{*}\right)}^{2}}$$

#### 2.10.2. Stability analysis

For stability analyses, yogurt samples were measured on a Turbiscan (Turbiscan^™^ LAB Stability Analyzer, Formulation SA, France) during 30 min. The global Turbiscan stability index (TSI) was reported. After refrigeration (4°C), yogurts were shaken and subsequently dispensed into the measurement vials of the equipment. The analyses were performed in triplicate for FY and in duplicate for controls.

#### 2.10.3. Flow behavior and viscoelasticity analyses

Flow behavior and viscoelasticity analyses were carried out on a rotational rheometer (HDR1, Thermal Analysis Instruments, DE, USA) at 4°C. For both tests a concentric cylinder geometry (28 × 42 mm) with a gap of 1,000 μm was used. For the flow behavior test, the yogurt viscosity was measured in a range of shear rates between 1 and 160 s^−1^. The data were fitted to the Ostwald–de Waele model and the viscosity of the yogurts was reported at a shear rate of 120 s^−1^. For the viscoelasticity test, a previous strain sweep was performed to find the linear viscoelastic region (LVR) in a range between 0.1 and 30% of oscillation strain. Then, the frequency sweep was performed at the LVR in a range of frequencies between 1 and 10 Hz. The FY was compared with UC and SC, which underwent the same tests. The G’ and G” values were reported at a 1 Hz frequency. The analyses were performed in triplicate for FY and in duplicate for controls.

### 2.11. Statistical analysis

The results were analyzed using the Minitab 18^®^ statistical program with a confidence level of 95%. For proximate analysis, the effect of pulp color was evaluated using the *t*-student test. An analysis of variance (ANOVA) was performed on the data of total carotenoids extracted by CE and UAE. Statistical significance was determined by applying the Tukey’s test for the CEB response variable from CE and for the three UAE response variables. Similarly, Tukey’s test was used to determine the statistical significance of the response variables related to the rheological and stability properties of yogurt.

## 3. Results and discussion

### 3.1. Chemical characterization of coffee pulp

Table 1 presents the results of the proximate analysis for red and yellow dehydrated pulp, showing differences in certain parameters between the two colors. Notably, the percentage of TDF was significantly higher in yellow pulp (68.52 ± 3.72%) as compared to red pulp (45.17 ± 1.07%) (*p* < 0.05). TDF and IDF values differed significantly (*p* < 0.05) between the two pulp colors. Despite these differences, the fiber values are close to those reported by Mindarti et al. (51), for Arabica brown pulp flour (63.16 ± 0.56% DW, 58.38 ± 0.23% DW, and 4.78 ± 0.33% DW of TDF, IDF and SDF respectively). These values are closer to those reported in this study for yellow pulp (68.52 ± 3.72% DW, 62.31 ± 3.14% DW, and 6.21 ± 0.58% DW of TDF, IDF and SDF respectively). Accordingly, the potential use of the pulp to take advantage of its fiber content is evident, both to produce foods that help the digestive system to function properly and to produce other food additives (51).

**Table 1:** Macronutrient composition of red and yellow coffee pulp (*Coffea arabica* var. Caturra).

Parameters ^a^	Red coffee pulp	Yellow coffee pulp
[%]		
Humidity (FM)	75.94 (2.17)	79.93 (0.30)
Lipids ^b^	3.91 (0.20)	3.99 (0.14)
Protein ^b^	9.67 (0.29)	9.44 (0.06)
Ashes ^b^	4.88 (0.01)	4.80 (0.01)
TC ^b^	77.15 (1.82)	77.54 (6.90)
TDF ^b^	45.17^B^ (1.07)	68.52^A^ (3.72)
IDF ^b^	38.11^B^ (0.55)	62.31^A^ (3.14)
SDF ^b^	7.06 (0.52)	6.21 (0.58)
Moisture (DW)	4.39 (0.19)	4.24 (0.03)

FM, Fresh mass; TC, Total carbohydrates; TDF, Total dietary fiber; IDF, Insoluble dietary fiber; SDF, Soluble dietary fiber; DW, Dry weight. ^a^ Values in parentheses are standard deviations, *n* = 3.^b^ Values reported on dry weight. Values with different capital letter between red and yellow samples are significantly different (*p* < 0.05) according to *t*-Student test.

The fat content of the red and yellow pulp in this study was found to be independent of the color (*p* > 0.05), with values of 3.91 ± 0.20% and 3.99 ± 0.14%, respectively. Such values represent differences of less than 20% as compared to 4.28 ± 0.01% and 4.10 ± 0.26% reported in other studies of coffee pulp (20, 52). In terms of ash content, the red and yellow pulp had values of 4.88 ± 0.01% and 4.80 ± 0.01%, respectively. These values are roughly 50% lower than the values reported by Patil et al. (53), for coffee pulp (9.6 ± 2.2%). Patil et al. (53) also revealed that pulp coffee contained high concentrations of minerals such as potassium (410 mg/100 g DW), calcium (97.8 mg/100 g DW), and magnesium (24.5 mg/100 g DW).

The present study determined a caffeine concentration of 8.81 ± 0.18 and 9.06 ± 0.17 mg/g DW for the red and yellow coffee pulps, respectively. Several studies have analyzed the caffeine content in coffee pulp, with varying results depending on the coffee species, the processing method, and the region of cultivation. For example, a study conducted in Mexico (54), reported caffeine levels of 18.60 ± 0.31 mg/g in fresh coffee pulp. Furthermore, another study investigated the caffeine content in six coffee cherry pulps from several *Coffea arabica* cultivars (Bourbon, Caturra, mixed varieties, Catuai, and Maragogype), origin (Salvador, Honduras, and Congo), and processing methods (wet and semi-dry depulping) (55). The results showed caffeine concentrations ranging from 3.4 to 6.8 mg/g in dried coffee pulp samples. The Caturra sample had a caffeine content of 4.6 mg/g according to Heeger et al. (55), which is half the value found in the present study for the same variety.

### 3.2. Total carotenoids content by UV–vis spectroscopy

Data on TCC obtained by CE and UAE methods using acetone are shown in Table 2. A comparison was made between the two drying methods (forced convention and freeze-dried) to determine the most appropriate approach for ultrasound-assisted extraction. Thus, a TCC of 1.06 ± 0.02 mg *all-trans*-β-carotene equivalents/g biomass DW for forced convection drying and 1.58 ± 0.08 mg *all-trans*-β-carotene equivalents/g biomass DW for freeze-drying was obtained for the red pulp; this implies an increase in carotenoids of 33% when the pulp is freeze-dried. For yellow pulp, a CEB of 1.19 ± 0.09 mg *all-trans*-β-carotene equivalents/g biomass DW was obtained for forced convection drying and 1.646 ± 0.004 mg *all-trans*-β-carotene equivalents/g sample DW for freeze-drying; this implies an increase in carotenoids of 28% for freeze-drying. Based on these comparisons, it is possible to define that UAE extractions should be carried out using freeze-dried pulp. For example Coelho et al. (56), extracted carotenoids from freeze-dried cashew apple using different solvents, including acetone, and achieved a concentration of 0.102 ± 0.017 mg β-carotene equivalents/g sample. For their part, Sánchez-Camargo et al. (57) performed an extraction of total carotenoids from mango peel dried by forced convection and freeze-drying using cold acetone and vortex agitation. They found a concentration similar in order of magnitude and value (1.22 ± 0.02 mg *all*-*trans*-β-carotene/g DW for forced convection and 2.01 ± 0.05 mg *all*-*trans*-β-carotene/g DW for freeze-dried) to those found in the present study. Furthermore, López-Bermúdez et al. (45) extracted carotenoids from freeze-dried tomato peel using acetone, which was able to extract 3.06 ± 0.33 mg β-carotene equivalents/g DW. According to these results, coffee pulp has a large potential to be a carotenoid source that can be used in industry and compete against currently established raw materials.

**Table 2:** Concentration of total carotenoids extracted with acetone using ultrasound and conventional extraction techniques.

Color	Drying method	Extraction method	Concentration (mg^a^/g sample)
Concentration (μg all-trans-β-carotene equivalents/g sample)
Red	Forced convection	CE	1.06 (0.02)
	Freeze-dried	CE	1.58 (0.08)	Ultrasound	1.72 (0.17)
	Yellow	Forced convection	CE	1.19 (0.09)
Freeze-dried		CE	1.646 (0.004)	Ultrasound	2.059 (0.001)

Values in parentheses are standard deviations. *n* = 3. mg^a^, mg of β-carotene equivalents/g sample. CE, Convectional Extraction.

### 3.3. Carotenoids profiling by RP-UHPLC-DAD

**Figure 2:**
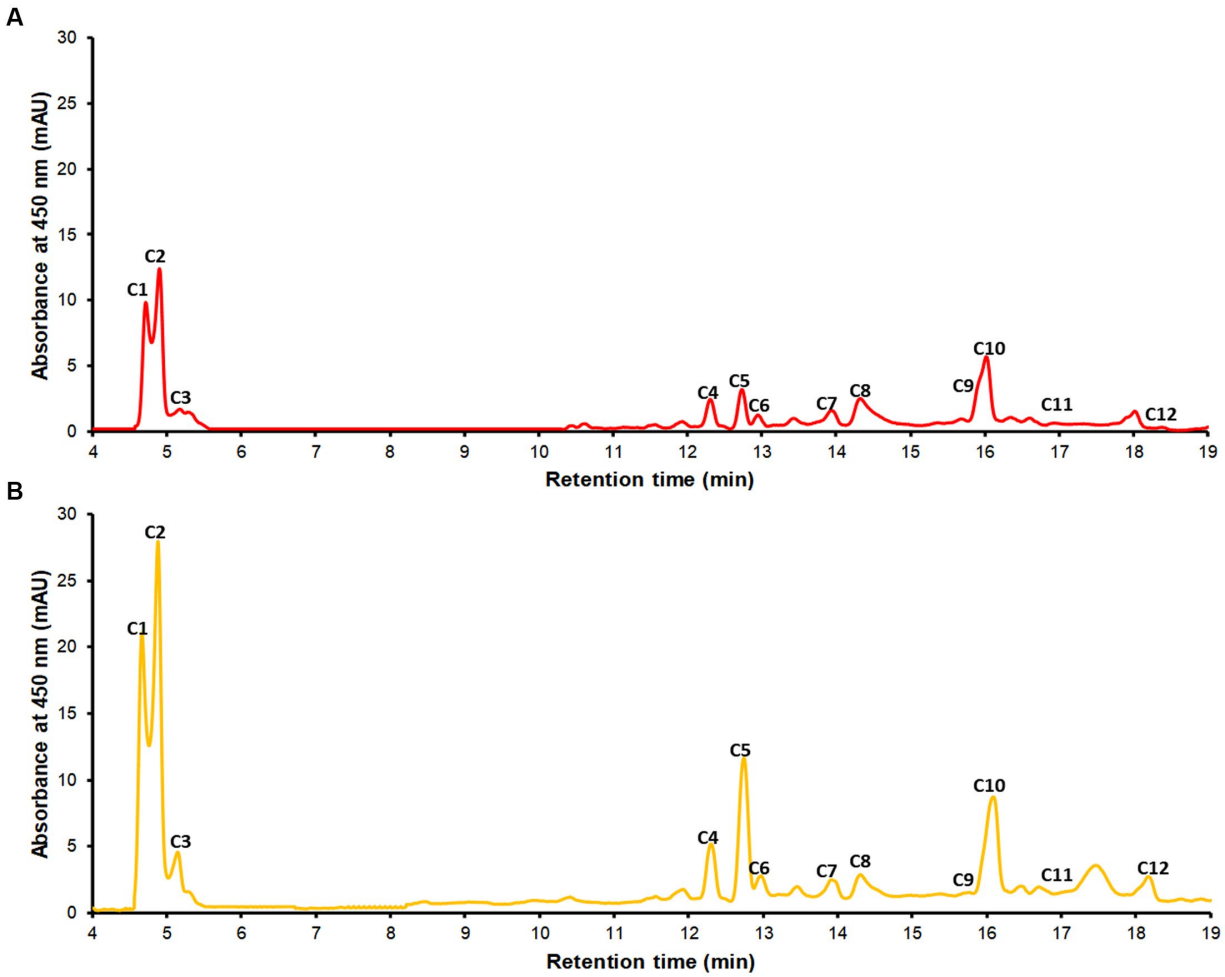
The chromatographic profile recorded at 450 nm of the extracts from red pulp **(A)** and yellow pulp **(B)** by RP-UHPLC-DAD. Identification of the peaks are described in Table 3.

The chromatographic profile at a wavelength of 450 nm of the extracts is shown in Figure 2. Tentatively, 12 carotenoids were identified in the samples, using their UV–Vis spectra, reference parameters [spectral fine structure (III/II) % and maximum cis intensity (II/B)], and in some cases comparison with authentic standards (Table 3). The coffee pulp residue samples showed a wide variety and concentration of xanthophyll esters. These esters have much longer retention times than their precursor xanthophylls because the resulting molecule contains a much larger hydrophobic (nonpolar) part and therefore are more retained in the nonpolar stationary phase. Additionally, based on the UV–Vis spectra of these esters, it was determined that they are all lutein derivatives (16, 21). The studied parameters, however, do not provide structural information beyond their precursor xanthophyll. To completely elucidate the structure, it would be necessary to perform chromatographic studies on alkaline hydrolysis products and analysis of the non-hydrolyzed extract using RP-UHPLC-APCI-MS (58). Yellow coffee pulp residues exhibited higher concentrations of each analyte. Both red and yellow coffee samples were mainly composed by lutein isomers (c1, c3) and lutein esters (c7–c12) at high concentrations (579.1 ± 21.3 and 808.0 ±18.3 μg *all-trans*-β-carotene equivalents/g sample for red and yellow coffee, respectively). Additionally, significant amounts of zeaxanthin (c2), β-carotene isomers (c6, c7, c8), and α-carotene (c5) were found, although the presence of violaxanthin and neoxanthin was also expected based on previous reports (21, 59–61).

**Table 3:** Identification and quantification of carotenoids in red and yellow coffee pulp by RP-UHPLC-DAD.

Peak no.	t_R_ (min)	λ_máx_ (nm)	(III/II)%	Ratio (II/B)	Tentative identification	Concentration (μg all-*trans*-β-carotene equivalents/g sample)^b^	
						Red coffee pulp	Yellow coffee pulp
c1	4.72	420, 446, 472	56	NA	*all*-*trans*-lutein^a^	182.5 (19.7)	327.0 (0.6)
c2	4.90	420, 449, 472	21	NA	*all*-*trans*-zeaxanthin^a^	252.6 (27.4)	475.2 (3.5)
c3	5.17	370, 422, 440, 467	40	2	13-*cis*-β-lutein	36.0 (5.4)	90.2 (2.2)
c4	12.30	425, 446, 475	50	NA	*all*-*trans*-α-carotene	57.1 (7.3)	89.5 (4.8)
c5	12.73	425, 453, 477	20	NA	*all*-*trans*-β-carotene^a^	176.2 (12.9)	229.0 (9.1)
c6	12.94	334, 419, 447, 470	10	2.3	13-*cis*-β-carotene	25.6 (3.6)	40.6 (3.5)
c7	13.93	427, 445, 472	NA	NA	Lutein ester	35.5 (5.6)	39.5 (5.5)
c8	14.32	426, 445, 473	NA	NA	Lutein ester	45.8 (1.6)	70.8 (6.6)
c9	15.89	425, 447, 474	NA	NA	Lutein ester	59.2 (3.3)	BLOQ
c10	16.01	422, 443, 474	NA	NA	Lutein ester	113.9 (9.5)	230.4 (27.8)
c11	16.86	421, 446, 472	NA	NA	Lutein ester	34.4 (4.5)	BLOQ
c12	18.18	420, 442, 474	NA	NA	Lutein ester	BLOQ	50.1 (7.2)

^a^ Identified using authentic standard. tR, λ_máx_ (nm), and (III/II)% values of the chromatographic peaks were identical to those of the authentic standards injected separatedly. ^b^ The presented results are the average of four replicates. Values in parentheses are standard deviations. Each compound was quantified by means of a calibration curve with *all*-*trans*- β-carotene. t_R_ (min), Retention time in min. NA, Not applicable. BLOQ, Below limit of quantification. LOQ was 1.0 μg/mL. Limit of detection (LOD) was 0.3 μg/mL.

### 3.4. Extraction of carotenoids using UAE

**Figure 3:**
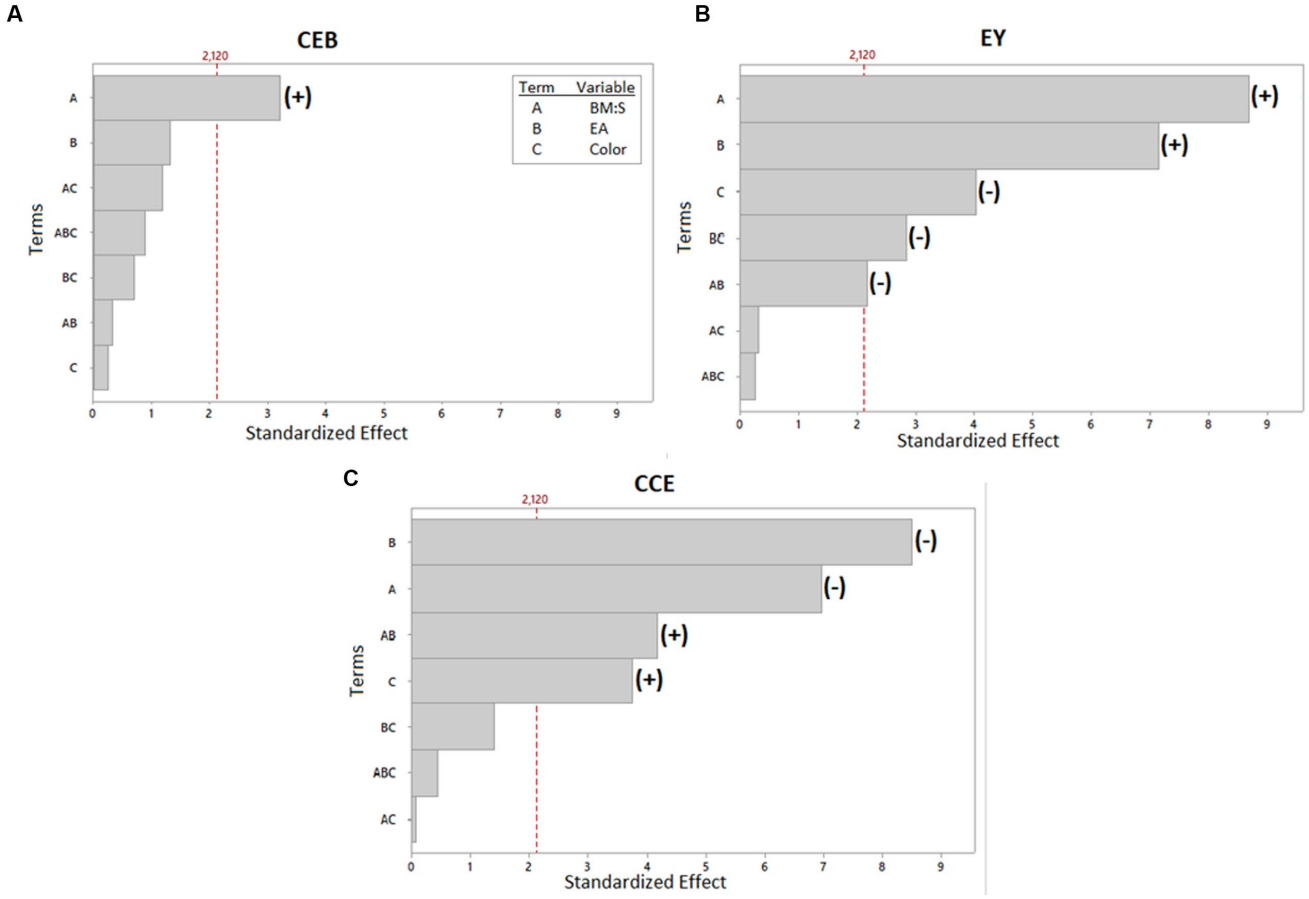
Pareto plot of standardized effects for the three response variables: **(A)** mg β-carotene equivalents/g biomass DW, CEB. **(B)** g extract/100 g biomass DW, EY. **(C)** mg of β-carotene equivalents/g extract, CCE. The terms which had significant effects were indicated with a (+) if it had a positive effect or with (−) if it had a negative effect.

Table 4 displays the concentrations obtained from the experimental UAE design. The ANOVA is found in Supplementary Material 1. It shows the model fit for the significant terms of each of the response variables. The *R*^2^ values for EY and CCE were high (90.59 and 89.32%, respectively) and low for CEB (33.36%). In the last case, only one term was significant (term A: BM:S), which could lead to this result. Similarly, the model had no lack-of-fit (*p* > 0.05), for the three response variables. The Pareto plot (Figure 3) shows with a significance level of 5%, the terms (variables or their interactions) that significantly impacted the CEB, EY, and CCE response variables. Some terms positively or negatively affected the response variables; these effects are also shown in the Pareto plot. The results indicate that, for both red and yellow pulp, the highest level of BM:S ratio (this is 1:40 *w/v* ratio) produced a higher CEB value. The relationship with the variable E:A ratio is not so clear, in fact, it was not significant according to the Pareto plot. The highest CEB was obtained for the extraction made with yellow pulp, with a BM:S ratio of 1:40 (*w/v*) and an E:A ratio of 20:80 (*v/v*) (2.30 ± 0.14 mg *all*-*trans*-β-carotene equivalents/g biomass DW); however, the statistical analysis shows that there were no significant differences in the results found for that response variable. For the response variable EY, the highest percent yields were found when the E:A ratio was more concentrated for ethanol; in addition, higher EY values were also obtained when the BM:S ratio level was higher (1:40 *w/v*). The highest EY (7.30 ± 0.51%) was obtained for the extraction made with red pulp, with a BM:S ratio of 1:40 *w/v* and an E:A ratio of 80:20 *v/v*. Lastly, CCE values were higher for the lowest BM:S ratio level (1:20 *w/v*) and for more concentrated E:A ratios in ethyl acetate (20:80, etanol:ethyl acetate, *v/v*). The best extraction configuration was taken based on this response variable and was achieved with the yellow pulp, a BM:S ratio of 1:20 *w/v* and an E:A ratio of 20:80 *v/v* to obtain 51.28 ± 4.52 mg *all-trans*-β-carotene equivalents/g extract DW. In comparison, Ordóñez-Santos et al. (62) used sunflower oil in UAE of carotenoids from mandarin peel and obtained 1.40 ± 0.02 mg β-carotene equivalents/g of sample, which is similar to the results obtained in this study. Likewise, Ordóñez-Santos et al. (27) performed carotenoid extractions from peach palm fruit peel using sunflower oil as solvent and obtained a maximum concentration of 1.63 ± 0.04 mg total carotenoids/g peel DW. According to the classification criteria proposed by Britton et al. (63), the coffee pulp used in this study is classified as “very high” for having a carotenoid concentration greater than 2 mg/100 g (230 ± 14 mg/100 g maximum for yellow pulp and 218 ± 2 mg/100 g maximum for red pulp).

**Table 4:** Experimental conditions and results for carotenoid extraction from red and yellow coffee pulp using ultrasound-assisted extraction.

Color	BM:S ratio^a^ (g/mL)	E:A ratio^b^ (*v/v*)	CEB^c^ (mg β-carotene eq./g biomass DW^f^)	EY^d^ (%, g extract/100 g biomass DW)	CCE^e^ (mg β-carotene eq./g extract)
Red	1:20	20:80	2.116^A^ (0.156)	4.315^D^ (0.003)	49.03^A^ (3.63)
	1:20	80:20	2.028^A^ (0.065)	6.251^AB^ (0.425)	32.55^BC^ (2.60)
	1:40	20:80	2.154^A^ (0.202)	5.986^BC^ (0.576)	36.00^BC^ (0.98)
	1:40	80:20	2.184^A^ (0.020)	7.302^A^ (0.507)	30.00^C^ (2.07)
Yellow	1:20	20:80	2.062^A^ (0.140)	4.026^D^ (0.083)	51.28^A^ (4.52)
	1:20	80:20	1.991^A^ (0.005)	5.121^CD^ (0.359)	39.02^B^ (2.85)
	1:40	20:80	2.300^A^ (0.146)	5.892^BC^ (0.417)	39.06^B^ (1.21)
	1:40	80:20	2.176^A^ (0.008)	6.192^ABC^ (0.436)	35.26^BC^ (2.55)

Values in parentheses are standard deviations. BM:S ratio^a^, Biomass: Solvent ratio; E:A ratio^b^, Ethanol: Ethyl acetate; CEB^c^, Carotenoids extracted from biomass; EY^d^, Extraction yield; CCE^e^, Carotenoids content in extract; DW^f^, Dry Weight. For the same column, values with different capital letters indicate statistically significant differences (*p* < 0.05) according to Tukey’s test *n* = 3.

Carotenoid extraction is generally higher when obtained by UAE than when obtained by the conventional method. For example, Coelho et al. (56) used UAE to extract carotenoids from red and yellow cashew apple shells using a mixture of acetone and methanol as solvent. They found in 11 of their 15 experiments a higher concentration of carotenoids when extracted by UAE as compared to conventional extraction. Thus, according to the concentrations obtained by UAE, this method proves to be striking for the extraction of carotenoids from plant material. This method has the potential to replace conventional extraction methods and, together with the use of environmentally friendly solvents, decrease the use of environmentally harmful methods.

### 3.5. *In vitro* antioxidant capacity assays of the carotenoid-enriched extract

Coffee pulp is a rich source of antioxidants and other bioactive compounds that have potential health benefits. Some compounds such chlorogenic acid isomers, anthocyanins, and carotenoids have been identified in Arabica coffee varieties with different external fruit color (16). As demonstrated by Geremu et al. (64), nevertheless, the type of solvent extraction exerts a significant influence on the type of bioactive compounds extracted from coffee pulp, and consequently on the antioxidant activity of the extracts. In this study, the optimal extract obtained may contain medium to non-polar compounds (such as β-carotene), since an 80:20 *v/v* solvent mixture of ethyl acetate:ethanol was used. The concentration (in μg/mL) of the UAE extract able to inhibit 50% of the DPPH free radical (EC_50_ value) was assessed. The results showed EC_50_ values of 19.34 ± 1.14 μg/mL. Similar values were found by Manasa et al. (11), in *Coffea canephora* (Robusta variety grown in India) pulp. Ice cold ethanol was used as solvent because the aim of that work was to extract polyphenols from coffee pulp, having an effective concentration (EC_50_) value of 12.75 μg/mL. In our study, the EC_50_ value of enriched-carotenoids extract was determined for the first time, and hence no literature data are available to compare our results with information from other literature. Additionally, the TEAC of the optimal UAE extract was measured using ABTS as radical and a value of 721.08 ± 51.23 μmol Trolox equivalent/g extract was achieved. In the same way, Thaiphanit et al. (20) reported comparable results for Arabica (710.43 ± 32.76 μmol/g) and Robusta (600.46 ± 25.07 μmol/g) coffee pulp extracts, which were obtained using 95% ethanol as the extraction solvent. Meanwhile, Corrêa-Filho et al. (65) measured the TEAC of neat β-carotene (before to encapsulate it) and they found values of 2,350 μmol Trolox equivalent/g β-carotene. This means that 3.26 g of the enriched-carotenoids coffee pulp extract obtained in this study, could have the antioxidant capacity equivalent to 1 g of β-carotene.

### 3.6. Encapsulation of optimal carotenoid-enriched extract by spray drying

The extract obtained by UAE with the highest CCE was chosen for encapsulation. The drying yield and encapsulation efficiency, as well as the powder characterization, are presented in Table 5, which can be directly compared with the study by López-Bermúdez et al. (45). The main difference between the two studies was found in the drying efficiency, which was 93.84 ± 1.08% in the present work, about 30% higher than that one achieved by López-Bermúdez et al. (45) (67.3 ± 0.5%). The main reason for encapsulating the extract is to protect it from degradation due to contact of the carotenoids with oxygen, light, and high temperature, among others. As indicated by Corrêa-Filho et al. (65), however, in the encapsulation process, the higher the air outlet temperature the higher the loss of these compounds. This fact is evidenced in the encapsulation efficiency values. In the present study, the encapsulation efficiency was 45.57 ± 4.03% (*w/w*), 21.5% lower than the value achieved by López-Bermúdez et al. (45) (58.1 ± 0.8%, *w/w*) who used an air outlet temperature of 100°C. In the same way, Guadarrama-Lezama et al. (66) achieved even higher encapsulation efficiencies (83.9 ± 1.5%) for carotenoids dissolved in sunflower oil as solvent for the extraction of carotenoids from paprika (*Capsicum annuum* L.) and used an air outlet temperature of 70°C. In contrast, this reduction in the air outlet temperature increases the moisture percentage of the sample. The moisture percentage achieved in this work (3.03 ± 0.26%) is close to that reported by López-Bermúdez et al. (45) (2.5 ± 0.1%), however Guadarrama-Lezama et al. (66), reports a higher moisture value (5.0 ± 0.1%) which can be explained by the lower outlet temperature that they used. The water activity reported in this study (0.25 ± 0.01) was higher than that achieved in other studies. In the case of Guadarrama-Lezama et al. (66), they found an a_w_ of 0.18 ± 0.01 for encapsulation with sunflower oil. For their part, Zhang et al. (67) reported values between 0.15 ± 0.01 and 0.22 ± 0.04, which are closer to those reported herein. Thus, these lows a_w_ values are remarkable since the enzymatic action is reduced and bacterial growth in the powder is avoided. The dissolution rate in this study (153.5 ± 2.8 s) was similar as compared to that achieved by López-Bermúdez et al. (45) (188.68 ± 5.51 s). These values were, however, higher (between 75 and 86%) than those reported by Zhang et al. (67), who obtained values between 25.5 ± 0.7 and 47.5 ± 3.5 s. Similarly, the same authors report tapped density values between 0.628 ± 0.018 and 0.689 ± 0.014 g/mL which were between 20 and 27% higher than that reported in this study (0.50 ± 0.01 g/mL), which could offer a potential advantage in terms of transport efficiency. In relation to provitamin A activity, a value of 12.57 ± 0.60 μg RAE/g encapsulated was calculated. Considering that 9.5 g of encapsulated were added to 100 mL of yogurt, a value of 119 μg RAE/100 mL of yogurt is expected. Such value is close to 120 μg RAE/100 mL of food, which according to the Colombian legislation is needed to declare a food “Excellent source” of vitamin A. Losing about 50% of the carotenoids in the encapsulation process can be considered a high cost. However, encapsulation of carotenoid-rich extracts allows new applications of the extract. One of the most powerful applications is its new ability to dissolve in aqueous matrices because when the extract is only dissolved in oil, this dissolution is more difficult. This ability facilitates the formulation of new foods or the enrichment of existing foods and, therefore, can have a positive impact on consumer health.

**Table 5:** Characteristics of carotenoid-rich encapsulates obtained through spray drying.

Property	Value^a^
Drying yield (%)	91.45 (0.01)
Encapsulation efficiency (%)	45.57 (4.03)
Moisture (%)	3.03 (0.26)
Water activity (a_w_)	0.25 (0.01)
Dissolution rate (s)	153.5 (2.8)
Tapped density (g/mL)	0.50 (0.01)
Provitamin A (RAE)	12.57 (0.60)

^a^ Values in parentheses are standard deviations. a_w_, Water activity; RAE, Retinol Activity Equivalents.

### 3.7. Dairy product characterization

#### 3.7.1. Colorimetry index

Table 6 shows the values of colorimetric the parameters L*, a*, b*, and ΔE against the control. L* is an index that represents the brightness of the product, whereas a* takes negative values when the sample is more greenish and positive values when it is more reddish. Similarly, b* takes negative values with bluish tones and positive values with yellowish tones (68). L* values of 84.67 ± 2.22 and 77.77 ± 1.55 were found for UC and FY, respectively. Statistical analysis indicated a significant difference between these two values (*p* < 0.05); more specifically, the value decreased by approximately 8% with the addition of the powder. For the a* index, the values were − 0.53 ± 0.15 and 2.53 ± 0.06 for UC and FY, respectively, which has a significant difference (*p* < 0.05), indicating that the yogurt went from having a slightly greenish tone to having a more reddish color in this case. The b* index showed values of 13.10 ± 0.95 for UC and 12.97 ± 0.25 for FY, which have no significant difference (*p* > 0.05). Thus, the yogurt presented yellowish coloration before and after the addition of the encapsulate. The last indicator, ΔE, which indicates the total color difference of the FY against the control was 7.6 ± 0.8. This value indicates that there is indeed a color change in the yogurt, which was greater than that reported by Suwannasang et al. (69), in a study in which they added Sacha Inchi oil encapsulated using freeze-drying (4.8 ± 0.1) and spray drying (0.7 ± 0.6). According to the above, the addition of the encapsulation normally generates a color change that in some cases depends on the drying conditions, the wall materials, and the extract to be encapsulated.

**Table 6:** Colorimetric parameters, stability (TSI) and rheological properties of yogurt enriched with the encapsulated extract.

Property	Sample	Value
Colorimetry index L*	UC	84.67* (2.22)
	FY	77.77* (1.55)
Colorimetry index a*	UC	−0.53* (0.15)
	FY	2.53* (0.06)
Colorimetry index b*	UC	13.10 (0.95)
	FY	12.97 (0.25)
ΔE	FY	7.6 (0.8)
Turbiscan^®^ stability index (TSI)	UC	0.233^a^ (0.026)
	SC	0.334^a^ (0.039)
	FY	0.375^a^ (0.055)
Viscosity [Pa. s] (at 120 s^−1^)	UC	0.202^a^ (0.024)
	SC	0.019^b^ (0.003)
	FY	0.037^b^ (0.003)
n	UC	0.585^a^ (0.007)
	SC	0.363^c^ (0.012)
	FY	0.423^b^ (0.020)
K (Pa. s^n^)	UC	1.582^a^ (0.249)
	SC	0.334^b^ (0.029)
	FY	0.501^b^ (0.070)
*R* ^2^	UC	0.9718
	SC	0.9755
	FY	0.9576
G’ [Pa] (at 1 Hz)	UC	0.095^b^ (0.028)
	SC	0.022^b^ (0.004)
	FY	0.466^a^ (0.157)
G” [Pa] (at 1 Hz)	UC	0.156^b^ (0.003)
	SC	0.054^b^ (0.006)
	FY	0.625^a^ (0.140)

UC, Unstirred Control; SC, Stirred Control; FY, Fortified Yogurt; ΔE, total color difference; n, Flow Index; K, Consistency Index; G’, Storage Modulus; G”, Loss Modulus. Values in parentheses are standard deviations *n* = 3 for FY and *n* = 2 for both controls. For data presented in pairs, if they have *, they present significant differences (*p* < 0.05) according to *t*-Student test. For the same response variable, values with different letters indicate statistically significant differences (*p* < 0.05) according to Tukey’s test (*n* = 2 for UC and SC; *n* = 3 for FY).

#### 3.7.2. Stability analysis

The TSI is a specific parameter used to compare and characterize physical stability. Thus, the lower the value of the TSI, the greater the stability of the sample. Table 6 shows the TSI values for UC, SC, and FY (0.233 ± 0.026, 0.334 ± 0.039, and 0.375 ± 0.055, respectively), which had no significant differences (*p* > 0.05) among them. In general, the product had low TSI values, which is a remarkable result. These values may be comparable with yogurts with high pectin concentration that were studied by Miyaji et al. (70), who in their study also reached values close to 1 with the highest pectin concentration in drinking yogurts. Thus, drinkable yogurts show good stability characteristics indicating that they are a good product for fortification with encapsulates.

#### 3.7.3. Flow behavior and viscoelastic analysis

**Figure 4:**
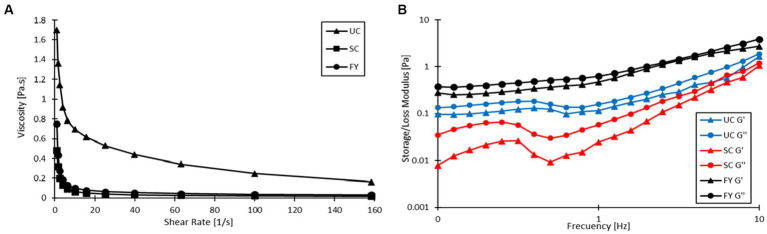
**(A)** Flow behavior of unstirred control (UC) stirred control (SC), and fortified yogurt (FY). **(B)** Viscoelastic properties of UC, SC, and FY.

Figure 4A shows the relationship between shear rate and viscosity, which reveals that all three products had a pseudoplastic behavior as viscosity decreased with increasing shear rate. As shown in Table 6, the viscosities of UC, SC, and FY were 0.202 ± 0.024, 0.019 ± 0.003 and 0.037 ± 0.003 Pa s, respectively, at 120 s^−1^. Statistically significant differences were found between the unstirred sample and the stirred samples (*p* < 0.05), this shows that the stirring process reduces the viscosity of the yogurt. Meanwhile, the sample with encapsulation compared to SC had no significant differences (*p* > 0.05) implying that the enrichment of the product *per se* does not alter this rheological property. Furthermore, de Campo et al. (71) also report a decrease in the viscosity of yogurt to which they added zeaxanthin nanoparticles (~0.6 Pa s) as compared to their control (~1 Pa s) without nanoparticle addition.

Table 6 also shows the fit of the flow behavior data to the Ostwald de Waele or Power Law model for the UC, SC, and FY samples. In the case of UC, the flow index (n) was 0.585 ± 0.007 which had significant differences (*p* < 0.05) with the n values of the other two yogurts. For the SC sample, the value of n was 0.363 ± 0.012, which was significantly lower (*p* < 0.05) as compared to UC, indicating that SC had a more shear thinning behavior than UC. For FY the n value was 0.423 ± 0.020 which is also statistically lower than UC (*p* < 0.05); moreover, it had also significant difference with SC (*p* < 0.05). This result reaffirms that the yogurt had a shear thinning behavior and may also be related to the fact that the change in yogurt viscosity may be more related to agitation than to the addition of the encapsulate itself. The consistency index (K) shows a different behavior. There was no statistically significant difference (*p* > 0.05) between K values for SC and FY (0.334 ± 0.029 and 0.501 ± 0.070 Pa s^n^, respectively). There was, however, a statistical difference between the K value of UC with respect to the two samples that were agitated (*p* < 0.05), which also leads to the fact that the viscosity decreased mainly with agitation.

Figure 4B shows the behavior of the elastic (storage, G’) and viscous (loss, G”) modulus as a function of frequency. The values of G’ (at 1 Hz) presented in Table 6 for UC, SC, and FY were 0.095 ± 0.028, 0.022 ± 0.004, and 0.466 ± 0.157 Pa, respectively. In this case, there was only significant difference (*p* < 0.05) between FY and the other two samples (UC and SC). In the case of G” (at 1 Hz), values of 0.156 ± 0.003, 0.054 ± 0.006, and 0.625 ± 0.140 Pa were obtained for UC, SC, and FY, respectively, and the statistical analysis showed significant differences again between FY and the UC and SC samples (*p* < 0.05). This is a remarkable behavior of the moduli and was also reported by Acharjee et al. (72), who argues that it may be related to the high-water holding capacity of the powder added to the yogurt. On the other hand, G’ values were always lower than G” and both moduli increased with increasing frequency, indicating that the yogurt has an elastic gel-like structure (73). According to the low values of the modulus, the gel-like structure is not very stable as compared to that reported by Acharjee et al. (72), (between 2,000 and 20,000 Pa) in a study where they enriched yogurt with orange pomace powder. This being so, the addition of the powder does not imply a significant change in the viscosity of the yogurt, as discussed above. Rather, the addition of the encapsulate may increase the gel-like elastic stability of the yogurt.

## 4. Conclusion

A comprehensive proximate analysis of coffee pulp was successfully conducted, revealing a high dietary fiber content that holds great potential in the food industry. Statistical analysis indicated significant differences (*p* < 0.05) in TDF and IDF between the two pulp colors. Freeze-drying proved effective in preventing carotenoid degradation, reducing it by up to 33% compared to forced convection drying. Furthermore, carotenoid extractions demonstrated that the BM:S ratio had the most significant impact on both the extracted carotenoid content and the extraction yield. RP-UHPLC-DAD analysis revealed that yellow pulp contained a higher concentration of carotenoids than red pulp. Among the extraction methods employed, the non-conventional method of ultrasound-assisted extraction (UAE) resulted in the highest concentration of extracted carotenoids, with a value of 2.30 ± 0.14 mg all-trans-β-carotene equivalents/g biomass DW. This value was 72% higher than that obtained using the conventional extraction method with acetone for the same pulp color. The encapsulation process demonstrated the efficiency of spray drying in retaining 45.57 ± 4.03% (w/w) of carotenoids in the powdered emulsion. Agitation during the mixing of the encapsulate into the dairy product led to a decrease in viscosity. The addition of the encapsulate had a low impact on the rheological properties, except for the elastic and viscous moduli (G’ and G”). The use of this encapsulate represents a potential advancement in the dairy industry as it allows for easy enrichment of products, even those that already have a significant vitamin A content. This study presents a promising solution for the utilization of coffee agro-industry by-products in Colombia and worldwide, addressing the large amount of waste generated in coffee-growing regions. Furthermore, this utilization contributes to reducing the environmental impact of industries, as the coffee pulp would no longer contaminate rivers and soil on farms.

## Data availability statement

The original contributions presented in the study are included in the article/supplementary material, further inquiries can be directed to the corresponding author.

## Author contributions

ER-O: investigation, writing—original draft, review, and editing. MH-C: conceptualization, supervision, writing—review and editing. JG-F: investigation. C-EN-C: review and editing. AS-C: conceptualization, supervision, visualization, resources, writing—original draft, review, and editing. All authors contributed to the article and approved the submitted version.

## Funding

AS-C expresses formal gratitude to the Vice Dean of Research at the Faculty of Engineering for the financial backing provided to this project via the Support Fund to Assistant Professors (FAPA). C-EN-C acknowledge the financial support given by Universidad Nacional de Colombia trough the project BIOREFINERIAS II: Estudio del potencial antioxidante y del contenido de fibra en biomasas no convencionales, con miras a explorar su aplicación en las industrias panificadora y aceitera, bajo la óptica del desarrollo sostenible, Hermes code 47233.

## Conflict of interest

The authors declare that the research was conducted in the absence of any commercial or financial relationships that could be construed as a potential conflict of interest.

## Publisher’s note

All claims expressed in this article are solely those of the authors and do not necessarily represent those of their affiliated organizations, or those of the publisher, the editors and the reviewers. Any product that may be evaluated in this article, or claim that may be made by its manufacturer, is not guaranteed or endorsed by the publisher.
