# Supplementary material for: Utilization of red and yellow Coffea arabica var. Caturra pulp: macronutrient analysis, carotenoid extraction, and encapsulation for dairy product enrichment
Source: Front Nutr. 2023 Aug 31;10:1231049. doi: 10.3389/fnut.2023.1231049 (PMC10501141; doi:10.3389/fnut.2023.1231049)
Supplement: Supplementary file 1 [file Table_1.docx]

Utilization of Red and Yellow Coffea arabica var. Caturra Pulp: Macronutrient Analysis, Carotenoid Extraction, and Encapsulation for Dairy Product Enrichment

Supplementary Material

**Elkin Rojas-Orduña, María Hernández-Carrión, Juan David Gómez-Franco, Carlos-Eduardo Narváez-Cuenca, Andrea del Pilar Sánchez-Camargo***

*** Correspondence:** Andrea del Pilar Sánchez-Camargo: [ad.sanchez@uniandes.edu.co](mailto:ad.sanchez@uniandes.edu.co)

**Supplementary Material 1.** Analysis of Variance of the three response variables

| **Response Variable** | **Source** | **DF** | **SS** | **MS** | **F-Value** | **p-Value** |
| --- | --- | --- | --- | --- | --- | --- |
| Content of carotenoids extracted from biomass (CEB) (mg β-carotene eq./ g DW) | Model | 1 | 0.14 | 0.14 | 11.01 | 0.003 |
|  | A: BM:S ratio | 1 | 0.14 | 0.14 | 11.01 | 0.003 |
|  | Lack of fit | 6 | 0.06 | 0.01 | 0.77 | 0.605 |
|  | Pure error | 16 | 0.22 | 0.01 |  |  |
|  | Total | 23 | 0.43 |  |  |  |
|  | R^2^ = 33.36% |  |  |  |  |  |
|  | R­^2^_adjusted_ = 20.69% |  |  |  |  |  |
| Extraction yield (EY) (%) | Model | 5 | 24.73 | 4.95 | 34.67 | 0.000 |
|  | A: BM:S ratio | 1 | 12.01 | 12.01 | 84.17 | 0.000 |
|  | B: E:A ratio | 1 | 8.10 | 8.10 | 56.75 | 0.000 |
|  | C: Color | 1 | 2.58 | 2.58 | 18.09 | 0.000 |
|  | A x B | 1 | 0.75 | 0.75 | 5.26 | 0.034 |
|  | B x C | 1 | 1.29 | 1.29 | 9.06 | 0.008 |
|  | Lack of fit | 2 | 0.03 | 0.01 | 0.09 | 0.913 |
|  | Pure error | 16 | 2.54 | 0.16 |  |  |
|  | Total | 23 | 27.30 |  |  |  |
|  | R^2^ = 90.59% |  |  |  |  |  |
|  | R­^2^_adjusted_ = 87.98% |  |  |  |  |  |
| Concentration of carotenoids in the extract (CCE) (mg β-carotene eq./ g extract) | Model | 4 | 1173.66 | 293.14 | 39.73 | 0.000 |
|  | A: BM:S ratio | 1 | 373.17 | 373.17 | 50.52 | 0.000 |
|  | B: E:A ratio | 1 | 557.08 | 557.08 | 75.42 | 0.000 |
|  | C: Color | 1 | 108.93 | 108.93 | 14.75 | 0.001 |
|  | A x B | 1 | 134.48 | 134.48 | 18.21 | 0.000 |
|  | Lack of fit | 3 | 17.06 | 5.687 | 0.74 | 0.545 |
|  | Pure error | 16 | 127.27 | 7.705 |  |  |
|  | Total | 23 | 1313.99 |  |  |  |
|  | R^2^ = 89.32% |  |  |  |  |  |
|  | R­^2^_adjusted_ = 87.07% |  |  |  |  |  |

DF: Degree of Freedom; SS: Sum of Squares; MS: Mean Squares. BM:S: Biomass: Solvent. E:A: Ethanol: Ethyl acetate.
